# Local ocular factors associated with the development of diabetic macular edema: an inter-eye study

**DOI:** 10.1038/s41598-023-42038-9

**Published:** 2023-09-08

**Authors:** Jaehwan Choi, Sang Jin Kim, Se Woong Kang, Ki Young Son, Sungsoon Hwang

**Affiliations:** 1 Department of Ophthalmology, Samsung Medical Center, Sungkyunkwan University School of Medicine, Seoul, South Korea; 2 Department of Ophthalmology, Chungnam National University Sejong Hospital, Sejong, South Korea

**Keywords:** Retinal diseases, Risk factors

## Abstract

To investigate local ocular factors associated with the development of diabetic macular edema (DME), we classified each eye of patients with unilateral DME as the DME eyes or the fellow eyes (without DME). We compared the clinical characteristics, optical coherence tomography (OCT), and OCT angiography (OCTA), ultra-wide field fundus photography, and angiography features of each eye. As a result, fifty-five patients with unilateral DME were enrolled. Although the diabetic retinopathy stage was not different between each group of eyes, DME eyes showed a higher prevalence of venous beading and a larger area of nonperfusion region than did fellow eyes (all *P* < 0.05). OCTA features of DME eyes also showed a larger foveal avascular zone in the deep capillary plexus and a lower vascular density in both the superficial and deep capillary plexuses (all *P* < 0.05). This study highlighted ocular features reflecting retinal ischemia, such as venous beading, area of nonperfusion region, and vascular density in the central retinal area, are associated with the development of DME. OCTA and ultra-wide field fluorescein angiography may be useful for evaluating the parameters of retinal ischemia and the risk of DME development.

## Introduction

Diabetic macular edema (DME) is one of the main causes of visual impairment in patients with diabetic retinopathy (DR). The prevalence of DME has been reported to vary among studies and diabetes type, ranging from 4.1 to 7.9% in type 1 diabetes and 1.4–12.8% in type 2 diabetes^1^. The pathophysiology of DME remains unclear. However, increased vascular permeability and breakdown of the blood-retinal barrier by hyperglycemia-induced oxidative stress and inflammation are considered key factors in the development of DME. Therefore, current treatment modalities for DME are carried out by suppressing cytokines of growth factor induced by inflammation and oxidative stress^2–6^.

Reported risk factors for DME include hyperglycemia, severity of DR, history of cataract surgery, duration of diabetes, puberty, pregnancy, hypertension, dyslipidemia, and nephropathy^7,8^. In addition, leptin, adiponectin, and genetic factors have been proposed as risk factors for DME^1^.

Although numerous studies have elucidated and proposed risk factors for DME, they have focused on systemic factors. This raises question whether any local ocular features are related to the development of DME. One way to determine this is to compare the ocular features between the eyes with DME and the eyes without DME in patients with unilateral DME. In this study, we conducted an inter-eye study of patients with unilateral DME to identify ocular features associated with the development of DME.

## Methods

### Setting

To identify local ocular factors related to the incidence of DME, we conducted a cross-sectional inter-eye comparison study of patients diagnosed with unilateral DME. In addition, the demographics and ocular features of patients with unilateral DME were compared with those of patients with bilateral DME. The study adhered to the tenets of the Declaration of Helsinki and was approved by the Institutional Review Board of the Samsung Medical Center, Seoul, South Korea (IRB number 2022-08-145). The board waived the need for informed consent owing to the retrospective design of this study.

### Subjects

**Figure 1:**
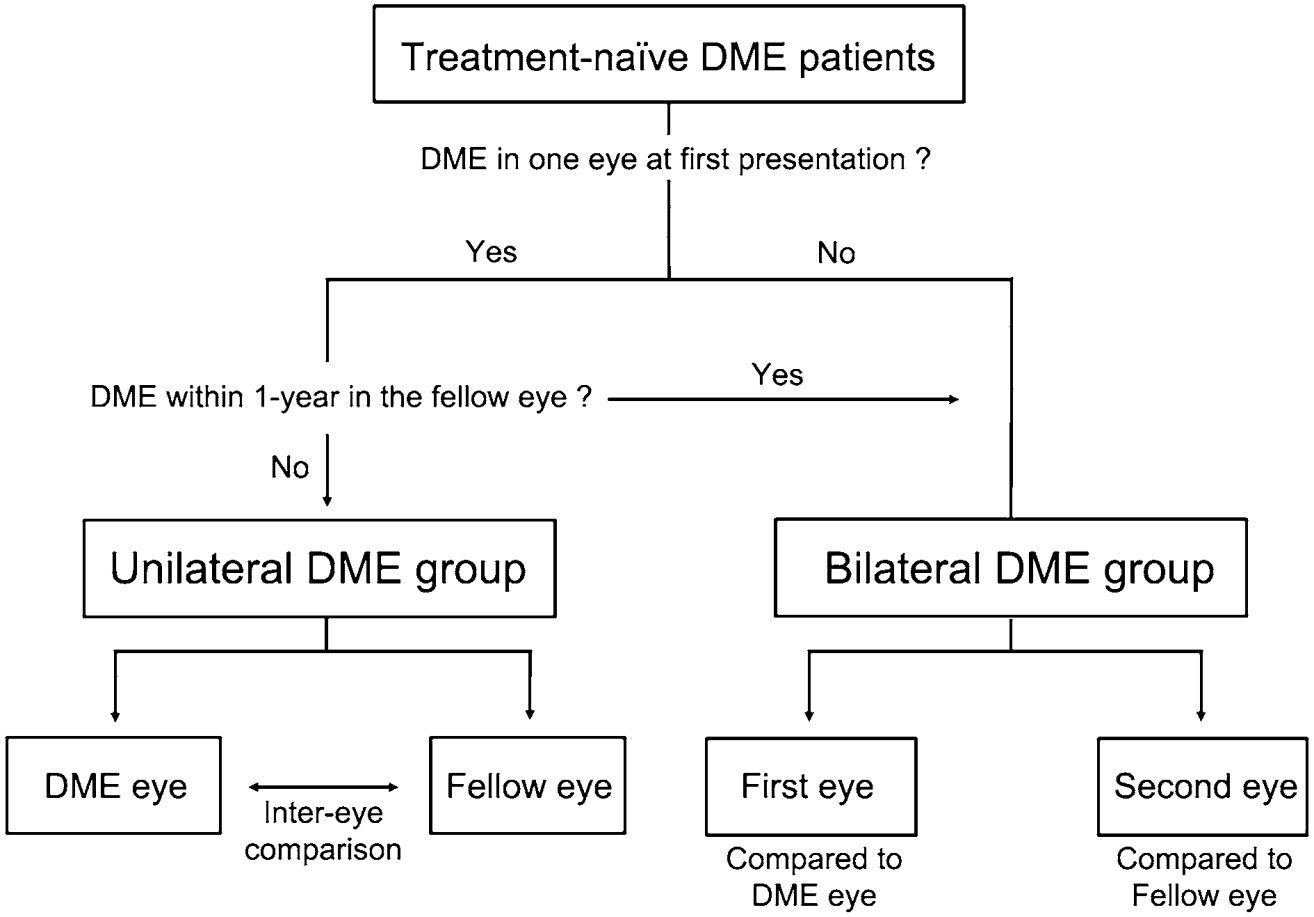
Flowchart describing the study selection process. DME, diabetic macular edema.

The electronic medical records of patients who were first diagnosed with DME between January 2018 and December 2020 at the Samsung Medical Center were retrospectively reviewed. DME was defined as the presence of DR and a central subfield thickness (CST) of > 300 μm on spectral-domain optical coherence tomography (OCT, Spectralis^®^; Heidelberg Engineering GmbH, Heidelberg, Germany). All enrolled patients were Korean. Patients with DME were divided into two groups: (1) patients with unilateral DME were those who had DME in one eye, and the fellow eye did not develop DME within 1 year; (2) patients with bilateral DME were those who had bilateral DME at initial presentation or unilateral DME at initial presentation, with the fellow eye developing DME within 1 year (Fig. 1). Patients with a history of ocular diseases such as retinal detachment, advanced glaucoma, or age-related macular degeneration, were excluded. Patients with a history of treatment for DR such as laser photocoagulation, intravitreal injection, or retinal surgery, were also excluded. To exclude pseudophakic cystoid macular edema, patients who underwent cataract surgery within 6 months were excluded.

### Examinations

We assessed demographic data including age, sex, type of diabetes mellitus (DM), hypertension, dyslipidemia, hemoglobin A1c (HbA1c) level, and effective glomerular filtration rate.

The eyes of patients with unilateral DME were divided into the DME eye and the fellow eye, on the basis of the presence of DME. The eyes of patients with bilateral DME were classified as the first eye (the eye with greater CST if bilateral DME was observed at the initial presentation or the eye in which DME occurred first) and the second eye (the eye with lesser CST or the eye in which DME occurred within 1-year after the first eye). Patients underwent ocular examinations, including best-corrected visual acuity, intraocular pressure, manifest refraction, noncontact tonometry, slit-lamp biomicroscopy, fundus examination, OCT, swept-source OCT angiography (OCTA, DRI OCT Triton; Topcon Corporation, Tokyo, Japan), and ultra-wide field fundus photography and fluorescein angiography (uWF-FP and uWF-FA, Optomap; Optos Plc, Dunfermline, Scotland, UK).

CST was then automatically measured, mean macular thickness in the central 1-mm diameter circle of the Early Treatment Diabetic Retinopathy Study grid^9^. We determined the stage of posterior vitreous detachment using OCT and OCTA images and classified it as one of five stages: from stage 0, i.e., vitreoretinal interface without posterior vitreous detachment, to stage 4, i.e., complete posterior vitreous detachment with release of vitreopapillary adhesion^10^. Superficial capillary plexus (SCP) and deep capillary plexus (DCP) images of the central 3 × 3 mm of the macula were automatically obtained by built-in OCTA software. We performed manual segmentation of OCT and OCTA images when automatic segmentation was improper. OCTA images with an image quality score less than 40 (ranging from 0 to 100 by built-in OCTA software) or those exhibiting motion artifacts, were excluded from the analysis. We calculated the vessel density (VD) of the SCP and DCP using AngioTool software (Angiotool 0.6a, https://ccrod.cancer.gov/confluence/display/ROB2) and area of the foveal avascular zone (FAZ) of the SCP and DCP using ImageJ software version 1.53 k (National Institutes of Health, Bethesda, MD, USA).

Two observers (J.C. and K.Y.S.), who were blinded to the clinical information, used uWF-FP and uWF-FA to evaluate the presence of venous beading, which was judged through additional discussions in case of inconsistency. Cohen’s kappa coefficient was calculated to evaluate interobserver reliability. The built-in software (Optos V2 Vantage Pro software; Optos Plc, Dunfermline, Scotland, UK) was used to project uWF-FA images stereographically, and the area of nonperfusion region was measured as the percentage of the nonperfusion area within the clearly observable retina on the frontal image of the uWF-FA. The area was measured using ImageJ software version 1.53 k by two blinded examiners (J.C. and S.H.), and the mean values were used.

### Statistical analyses

The Wilcoxon signed-rank test was used to compare paired continuous variables between the DME eye and the fellow eye. The McNemar–Bowker test was used to compare the paired categorical variables between the two groups.

Additional statistical analyses were performed to compare patients with unilateral and bilateral DME. The same number of patients as those with unilateral DME were randomly selected, and the same demographics and ophthalmic features as those of patients with unilateral DME were analyzed. Demographic data from patients with bilateral DME were compared with those with unilateral DME. The ophthalmic features at the time of study enrollment were compared between the DME eye of unilateral DME and the first eye of bilateral DME, and between the fellow eye of unilateral DME and the second eye of bilateral DME. Mann–Whitney U test was used to compare continuous variables, and Chi-squared test or Fisher’s exact test was used to compare categorical variables.

Statistical analyses were performed with R software (version 4.2.0; R Core Team (2022). R: A language and environment for statistical computing. R Foundation for Statistical Computing, Vienna, Austria. URL https://www.R-project.org). Statistical significance was set at *P* < 0.05.

## Results

### Patients with unilateral and bilateral DME

We compared the demographic and ophthalmic clinical characteristics of patients with unilateral DME and those with bilateral DME. During the same study period, 55 patients met the inclusion criteria for unilateral DME, and 110 patients met the criteria for bilateral DME. Therefore, in our tertiary hospital, the proportion of unilateral DME was approximately 33.3% of the total incidence of DME cases. Of the 110 patients with bilateral DME, 55 patients were randomly selected and their demographic and ophthalmic features were compared with those with unilateral DME. Table 1 shows the demographic and clinical characteristics of patients with unilateral and bilateral DME. The mean age of the patients with bilateral DME was 56.4 ± 11.5 years, which was younger than that of patients with unilateral DME (63.4 ± 8.8 years, *P* = 0.002). HbA1c level of the bilateral DME group was 8.79 ± 1.94% and it was significantly higher than that of the unilateral DME group (7.82 ± 1.43%, *P* = 0.021). However, there were no differences in the type of DM, effective glomerular filtration rate, and the ratio of hypertension and dyslipidemia between the unilateral and bilateral groups (*P* = 1.000, 0.191, 0.703, and 0.340, respectively).

**Table 1:** Demographic characteristics of patients with unilateral and bilateral DME.

	Unilateral DME	Bilateral DME	*P* value
Age, y (SD)	63.42 (8.76)	56.42 (11.52)	0.002*
Sex, M : F	30: 25	30: 25	1.000
DM duration, y (SD)	17.0 (10.6)	14.6 (9.7)	0.287
DM type, n (%)			1.000
Type 1 DM	1 (1.8%)	2 (3.6%)	
Type 2 DM	54 (98.2%)	53 (96.4%)	
HbA1c, % (SD)	7.82 (1.43)	8.79 (1.94)	0.021*
eGFR, mL/min (SD)	82.8 (25.5)	88.6 (24.3)	0.191
HTN, n (%)	27 (49.1%)	30 (54.5%)	0.703
Dyslipidemia, n (%)	30 (54.5%)	24 (43.6%)	0.340

*DME* diabetic macular edema, *y* year, *SD* standard deviation, *M* male, *F* female, *DM* diabetes mellitus, *n* number, *HTN* hypertension.

*Statistically significant at *P*-value < 0.05.

### Inter-eye comparison in patients with unilateral DME

Table 2 shows the local ocular features and their comparison between DME eyes and fellow eyes. The mean best-corrected visual acuity of DME eyes was 0.22 ± 0.20 logarithm of the minimum angle of resolution (logMAR) (20/32) and 0.05 ± 0.08 logMAR (20/25) in fellow eyes (*P* < 0.001). The stage of DR was not significantly different between DME eyes and fellow eyes (*P* = 0.536). The mean CST of DME eyes was 362.6 ± 89.5 μm and was thicker than that of fellow eyes, which was 279.2 ± 32.8 μm (*P* < 0.001). Thirteen (23.6%) eyes in DME eyes and 10 (18.2%) eyes in fellow eyes were pseudophakia and the lens status was not different between two groups (*P* = 0.450).

**Table 2:** Inter-eye comparison of patients with unilateral and bilateral DME.

	Patients with unilateral DME		Patients with bilateral DME		*P*-value		
	DME eye	Fellow eye	First eye	Second eye	DME eye versus Fellow eye	DME eye versus First eye	Fellow eye versus Second eye
BCVA, Snellen (range), logMAR (SD)	20/32 (20/100–20/20), 0.22 (0.20)	20/25 (20/50–20/20), 0.05 (0.08)	20/32 (20/400–20/20) 0.27 (0.29)	20/30 (20/125–20/20) 0.18 (0.20)	< 0.001*	0.796	< 0.001*
Lens status, n (%)					0.450	1.000	1.000
Phakia	42 (76.4%)	45 (81.8%)	43 (78.2%)	46 (83.6%)			
Pseudophakia	13 (23.6%)	10 (18.2%)	12 (21.8%)	9 (16.4%)			
DR stage, n (%)					0.536	< 0.001*	< 0.001*
No DMR	0 (0.0%)	0 (0.0%)	0 (0.0%)	0 (0.0%)			
Mild NPDR	6 (10.9%)	9 (16.4%)	0 (0.0%)	0 (0.0%)			
Moderate NPDR	13 (23.6%)	15 (27.3%)	6 (10.9%)	9 (16.4%)			
Severe NPDR	35 (63.6%)	28 (50.9%)	24 (43.6%)	23 (41.8%)			
PDR	1 (1.8%)	3 (5.5%)	25 (45.5%)	23 (41.8%)			
PVD stage, n (%)					1.000	0.124	0.370
Stage 0	39 (70.9%)	40 (72.7%)	39 (70.9%)	40 (72.7%)			
Stage 1	9 (16.4%)	9 (16.4%)	7 (12.7%)	6 (10.9%)			
Stage 2	2 (3.6%)	1 (1.8%)	4 (7.3%)	5 (9.1%)			
Stage 3	1 (1.8%)	2 (3.6%)	5 (9.1%)	3 (5.5%)			
Stage 4	4 (7.3%)	3 (5.5%)	0 (0.0%)	1 (1.8%)			
AXL, mm (SD)	23.4 (0.8)	23.4 (0.8)	23.7 (0.9)	23.7 (0.9)	0.778	0.565	0.415
CST, μm (SD)	362.6 (89.5)	279.2 (32.8)	414.5 (117.0)	319.9 (49.8)	< 0.001*	0.012*	< 0.001*
Venous beading, n (%)	23 (41.8%)	12 (21.8%)	29 (52.7%)	23 (41.8%)	< 0.001*	0.340	0.006*
Area of nonperfusion region, % (SD)	10.4 (12.0)	6.3 (9.0)	14.0 (16.8)	11.2 (13.8)	0.004*	0.550	0.038*
SCP FAZ area, mm^2^ (SD)	0.41 (0.09)	0.38 (0.09)	0.51 (0.16)	0.40 (0.16)	0.190	0.088	0.706
DCP FAZ area, mm^2^ (SD)	0.81 (0.20)	0.67 (0.20)	0.80 (0.25)	0.84 (0.21)	0.013*	0.763	0.049*
SCP VD, % (SD)	48.8 (5.1)	52.7 (3.6)	47.6 (4.8)	47.5 (5.8)	0.008*	0.598	0.031*
DCP VD, % (SD)	55.9 (3.8)	59.3 (4.2)	53.5 (4.1)	53.5 (5.1)	0.001*	0.213	0.019*

*DME* diabetic macular edema, *BCVA* best corrected visual acuity, *logMAR* logarithm of the minimum angle of resolution, *SD* standard deviation, *n* number, *DR* diabetic retinopathy, *NPDR* non-proliferative diabetic retinopathy, *PDR* proliferative diabetic retinopathy, *PVD* posterior vitreous detachment, *AXL* axial length, *CST* central subfield thickness, *SCP* superficial capillary plexus, *DCP* deep capillary plexus, *VD* vascular density, *FAZ* foveal avascular zone.

*Statistically significant at *P*-value < 0.05.

Venous beading was observed in 23 (41.8%) eyes of DME eyes and 12 (21.8%) eyes of fellow eyes (*P* < 0.001). All patients who had venous beading in their fellow eyes had venous beading in their DME eyes. Cohen's kappa coefficient was 0.82, indicating a good agreement with the presence of venous beading. The mean area of nonperfusion region in DME eyes was 10.4 ± 12.0%, significantly larger than that of fellow eyes (6.9 ± 9.0%) (*P* = 0.004).

In the analysis of the OCTA images, the mean VDs in both the SCP and DCP were lower in DME eyes than in fellow eyes. The SCP VD was 48.8 ± 5.1% in DME eyes and 52.7 ± 3.6% in fellow eyes (*P* = 0.008). The DCP VD was 55.9 ± 3.8% in DME eyes and 59.3 ± 4.2% in fellow eyes (*P* = 0.001). The mean SCP FAZ area was 0.41 ± 0.09 mm^2^ in DME eyes and 0.38 ± 0.09 mm^2^ in fellow eyes (*P* = 0.190). The mean DCP FAZ was 0.81 ± 0.20 mm^2^ in DME eyes and 0.67 ± 0.20 mm^2^ in fellow eyes (*P* = 0.013).

### Comparison between patients with unilateral DME and patients with bilateral DME

**Figure 2:**
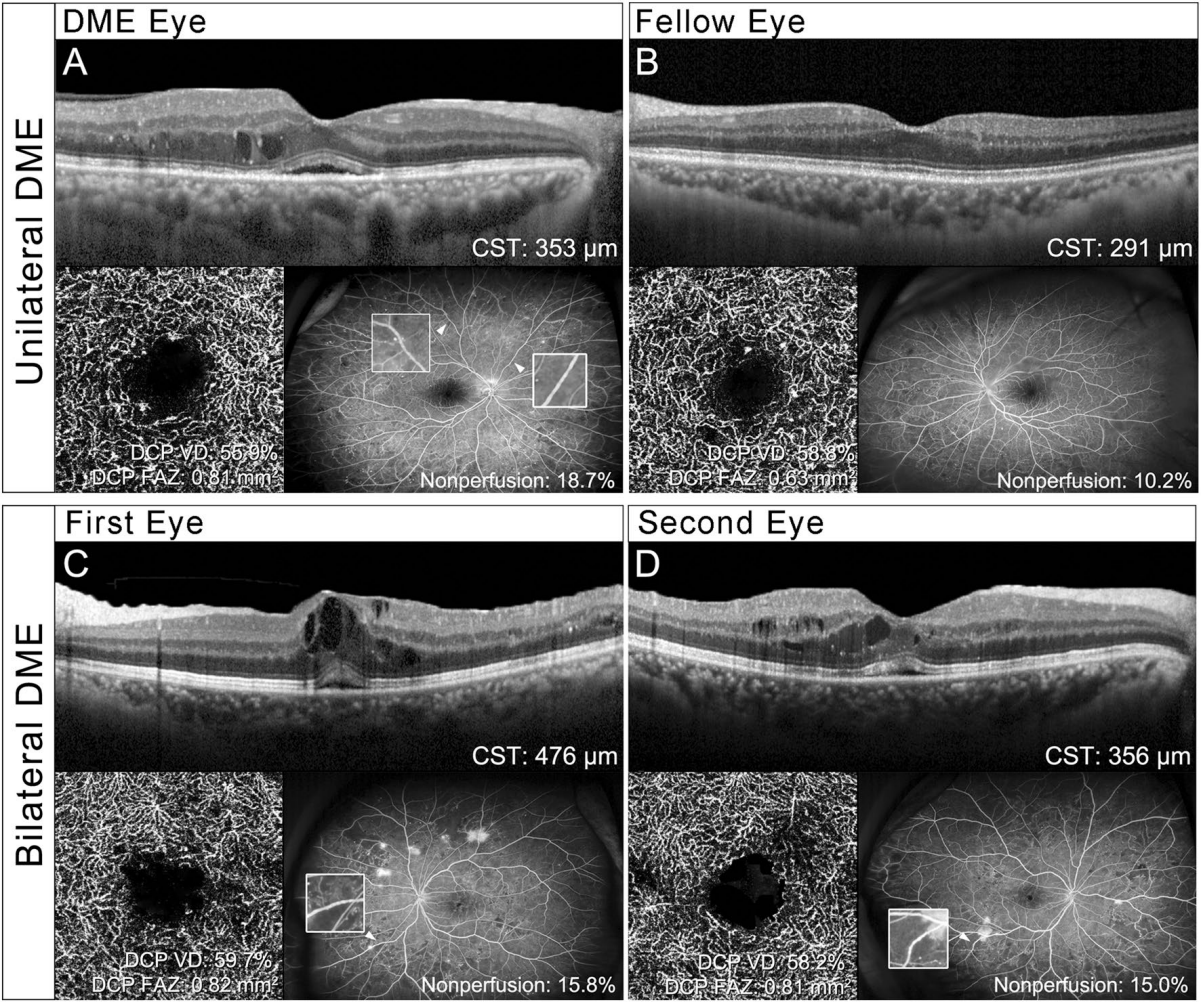
Representative unilateral and bilateral diabetic macular edema (DME) cases. (**A**, **B**) The patient with unilateral DME. A 67-year-old male with severe non-proliferative diabetic retinopathy in both eyes. (**A**) The DME eye shows intraretinal cysts and subretinal fluid. compared with the fellow eye (**B**), the DME eye has a larger foveal avascular zone and capillary dropout in the central 3 × 3-mm retina at the deep capillary plexus (DCP) in the optical coherence tomography angiography (OCTA) image. The area of nonperfusion region assessed by ultra-wide field fluorescein angiography shows that the DME eye has a larger area of nonperfusion region than does the fellow eye. Venous beading was present in the DME eye (white arrowhead), whereas it was not observed in the fellow eye. The image within the white border is a magnified picture of venous beading. (**C**, **D**) Patients with bilateral DME. A 53-year-old female with PDR in both eyes. Both eyes show intraretinal cysts and subretinal fluid. OCTA images of the DCP show an enlarged FAZ in both eyes. Retinal neovascularization is observed in both eyes and the area of nonperfusion region observed on fluorescein angiography is almost equal between (**C**) the first eye and (**D**) the second eye. Venous beading was present in both eyes (white arrowhead). CST, central subfield thickness; VD, vascular density.

The distribution of the DR stage showed a higher ratio of proliferative DR (PDR) in first eyes than in DME eyes and in second eyes than in fellow eyes (*P* < 0.001). In other words, patients with bilateral DME had a more advanced stage of DR. The ratio of venous beading was not significantly different between DME eyes in the unilateral DME group and first eyes in the bilateral DME group (*P* = 0.340). However, second eyes (41.8%) showed a higher ratio of venous beading than did fellow eyes (21.8%) (*P* = 0.006). Although the mean area of nonperfusion region was not significantly different between DME eyes and first eyes, it was significantly different between second eyes (11.2 ± 13.8%) and fellow eyes (6.3 ± 9.0%) (*P* = 0.038). None of the OCTA features differed significantly between DME eyes and first eyes. However, between fellow eyes and second eyes, the mean SCP VD (52.7 ± 3.6% and 47.5 ± 5.8%, *P* = 0.031) and DCP VD (59.3 ± 4.2% and 53.5 ± 5.1%, *P* = 0.019) were lower and the DCP FAZ area (0.67 ± 0.20 mm^2^ and 0.84 ± 0.21 mm^2^, *P* = 0.049) was larger in second eyes (Table 2). Representative cases from the unilateral and bilateral DME groups are shown in Fig. 2.

## Discussion

Although many studies have reported on DME, none have focused on unilateral DME. Unilateral DME accounted for one-third of all treatment-naïve patients with DME in the current study. In patients with unilateral DME, the mean best-corrected visual acuity of DME eyes was significantly worse than that of fellow eyes. This result is consistent with the fact that DME is one of the major causes of visual disturbance in patients with DR^11^.

The DR stage and a history of cataract surgery are known ocular risk factors for DME^12,13^. However, in the current study, the stage of DR between DME eyes and fellow eyes was not significantly different; severe non-proliferative DR (NPDR) was the most frequent, followed by moderate NPDR, mild NPDR, and PDR in both eyes. Because systemic factors are strongly associated with DR progression^1^, this may explain why there were no differences in the DR stage between the patients with unilateral DME. This study is worthwhile because it eliminated the effect of systemic factors and focused only on the local ocular features associated with the development of DME. However, caution is required when interpreting the result that DR stage was not different between DME eyes and fellow eyes. In this study, PDR represented 3.6% in unilateral DME eyes, whereas 43.6% in bilateral DME eyes. Therefore, it clearly showed a correlation between the severity of DR and the occurrence of bilateral DME. Our study indicates that in patients with unilateral DME, who were frequently in the NPDR stage, there was no difference in the DR stage between the two eyes. Therefore, the traditional DR staging system based on funduscopic findings^14^ has limitations in evaluating the risk of DME occurrence in patients with NPDR.

Systemic factors, including serum glucose levels, blood pressure, the presence of dyslipidemia, and renal function have been reported to be associated with the development of DME^1,7,8,15,16^. In this study, HbA1c level was higher in patients with bilateral DME compared to those with unilateral DME. However, no significant differences were found in other factors between two groups. All the patients enrolled in this study were diagnosed with DME. This could elucidate the different results in comparison to prior studies that compared patients with DME to those without DME^1,7,15,16^. Nonetheless, the result of this study highlights that serum glucose level is an important systemic factor associated with the development of DME, consistent with findings from previous studies^1,7,8^.

Vascular characteristics, including venous beading, area of nonperfusion region, SCP VD, DCP VD, and DCP FAZ area, showed that DME eyes had more ischemic changes than did fellow eyes. Venous beading is a focal venous change caused by retinal ischemia and is one of the major risk factors for DR progression^9,17^. In this study, 41.8% of DME eyes had venous beading, which was twice as common as that in fellow eyes. In previous studies, the prevalence of venous beading in patients with DR was reported to be 2.1–22.1%, and one study reported that venous beading was prominent in PDR (41.3%) but not in severe NPDR (5.9%) and moderate NPDR (0.0%)^17–19^. Compared with these results, the prevalence of venous beading in the DME eyes in our study was prominently high considering that all eyes except one eye (98.2%) were NPDR. This result suggests that DME eyes suffered from more ischemic changes than did those without DME in the same stages of DR.

Although increased area of retinal nonperfusion region outside the macula is related to the risk of DR progression^20^, the relationship with the development of DME is inconsistent^21–23^. In our study, DME eyes had larger nonperfusion areas observed in uWF-FA than did fellow eyes. Fang et al.^22^ suggested that not all nonperfusion areas identified in uWF-FA contribute to the development of DME, but viable cells near the nonperfusion area may produce more vascular endothelial growth factors, also known as vasopermeability factors, and other cytokines that promote vascular leakage and DME. Additional studies on the association with the development of DME according to the location or characteristics of nonperfusion are needed.

This study demonstrated that as well as the perfusion state outside the macula, the perfusion state of the central retina is also associated with the development of DME. Previous studies using OCTA showed that eyes with DME had a larger FAZ area and lower macular vascular density than did eyes without DME and normal eyes^24,25^. In this study, VDs in both SCP and DCP were lower in DME eyes. DCP FAZ was greater in DME eyes, but SCP FAZ was not. Our findings may support those of previous studies, which reported that DCP changes are more prominent than SCP changes in patients with DME, and DCP changes are related to anti-vascular endothelial growth factor response, photoreceptor recovery, and visual outcome after treatment^24,26^. DCP supplies the inner nuclear layer and outer nuclear layer, which are the main cystic areas in DME development^27,28^, and this may explain the different results between SCP FAZ and DCP FAZ in this study.

However, the aforementioned studies on fluorescein angiography and OCTA features of DME, did not control the stage of DR and systemic factors. In the unilateral DME patients, in which the stage of DR and systemic factors were controlled, the retinal ischemic features observed on fluorescein angiography and OCTA were significantly related to the development of DME. These can be used as ocular biomarkers to predict the risk of DME development. In patients who first present with unilateral DME, a risk of developing DME in their fellow eyes within 1 year is present if their fellow eyes have retinal ischemic features similar to those of their eyes with DME. In particular, although there are no differences in the stage of DR determined by funduscopic examination, angiographic features may help identify the risk of DME. In this regard, the value of OCTA is highlighted owing to its noninvasive nature.

In the comparison between patients with unilateral DME and those with bilateral DME, the types of DMs, presence of hypertension and dyslipidemia were not different. However, patients with bilateral DME were younger, had higher HbA1c level, and had a more advanced DR stage; the ratio of PDR was > 40% in both eyes of patients with bilateral DME. This implies that DME is likely to develop in both eyes if patient has advanced DR and poorly controlled diabetes at a young age. In contrast, DME is likely to occur in only one eye in older patients with a slow progression of DR. Vascular characteristics, including venous beading, area of nonperfusion region, FAZ, and VD, did not show differences between DME eyes of patients with unilateral DME and first eyes of patients with bilateral DME. However, comparing fellow eyes of patients with unilateral DME and second eyes of patients with bilateral DME, the capillary dropout assessed by the SCP VD, DCP VD, and DCP FAZ area was larger in second eyes. The comparison results were repeated identically to the comparison between the DME eyes and the fellow eyes. Therefore, these results reinforce our belief that the ischemic change detected on OCTA in the central retinal area is associated with the development of DME.

There are a few studies demonstrated that cataract surgery is risk factor of the DME development^7,13,29,30^. In the current study, however, there was no association between the history of cataract surgery and the development of DME. Because pseudophakic cystoid macular edema is common complication of cataract surgery in DR patients^31^, edema developed shortly after cataract surgery could be difficult to accurately distinguish whether it was DME, pseudophakic cystoid macular edema, or a potential overlap of the two conditions. Therefore, this study excluded patients underwent cataract surgery within 6 months to increase diagnostic accuracy of DME and to accurately analyze the local ocular factor for DME.

This study had several limitations. It was a retrospective cross-sectional study; therefore, concern about reverse causation is present. A potential of selection bias was present because the study population was based on patients who visited a single tertiary care center. Many patients with DME who visited the clinic during the study period and previously underwent treatment for DR or DME were excluded from this study. It was not possible to assess the OCTA images of all patients owing to the low image quality and artifacts.

In conclusion, unilateral DME is frequently found in older patients with slow progression of DR. Although there are no differences in the stage of DR between eyes, unilateral DME could be found if there is a difference in capillary nonperfusion, i.e., retinal ischemia between eyes. Vascular features obtained from fluorescein angiography and OCTA can be reliable biomarkers for evaluating the risk of DME development. In this regard, OCTA would be a useful measure to assess the risk of DME development owing to its noninvasive properties.

## Data Availability

The datasets generated and/or analyzed during the current study are available from the corresponding author on reasonable request.
